# Inhibition of Biofilm Formation, Quorum Sensing and Infection in *Pseudomonas aeruginosa* by Natural Products-Inspired Organosulfur Compounds

**DOI:** 10.1371/journal.pone.0038492

**Published:** 2012-06-08

**Authors:** Nathaniel C. Cady, Kurt A. McKean, Jason Behnke, Roman Kubec, Aaron P. Mosier, Stephen H. Kasper, David S. Burz, Rabi A. Musah

**Affiliations:** 1 College of Nanoscale Science and Engineering, State University of New York at Albany, Albany, New York, United States of America; 2 Department of Biological Sciences, State University of New York at Albany, Albany, New York, United States of America; 3 Department of Applied Chemistry, University of South Bohemia, Czech Republic; 4 Department of Chemistry, State University of New York at Albany, Albany, New York, United States of America; Vrije Universiteit Brussel, Belgium

## Abstract

Using a microplate-based screening assay, the effects on *Pseudomonas aeruginosa* PAO1 biofilm formation of several *S*-substituted cysteine sulfoxides and their corresponding disulfide derivatives were evaluated. From our library of compounds, *S*-phenyl-L-cysteine sulfoxide and its breakdown product, diphenyl disulfide, significantly reduced the amount of biofilm formation by *P. aeruginosa* at levels equivalent to the active concentration of 4-nitropyridine-*N-oxide* (NPO) (1 mM). Unlike NPO, which is an established inhibitor of bacterial biofilms, our active compounds did not reduce planktonic cell growth and only affected biofilm formation. When used in a *Drosophila-*based infection model, both *S*-phenyl-L-cysteine sulfoxide and diphenyl disulfide significantly reduced the *P. aeruginosa* recovered 18 h post infection (relative to the control), and were non-lethal to the fly hosts. The possibility that the observed biofilm inhibitory effects were related to quorum sensing inhibition (QSI) was investigated using *Escherichia coli*-based reporters expressing *P. aeruginosa lasR* or *rhIR* response proteins, as well as an endogenous *P. aeruginosa* reporter from the *lasI/lasR* QS system. Inhibition of quorum sensing by *S*-phenyl-L-cysteine sulfoxide was observed in all of the reporter systems tested, whereas diphenyl disulfide did not exhibit QSI in either of the *E. coli* reporters, and showed very limited inhibition in the *P. aeruginosa* reporter. Since both compounds inhibit biofilm formation but do not show similar QSI activity, it is concluded that they may be functioning by different pathways. The hypothesis that biofilm inhibition by the two active compounds discovered in this work occurs through QSI is discussed.

## Introduction

Biofilm formation by many pathogens is intimately linked to a form of inter-bacterial communication known as quorum sensing (QS), in which small diffusible signaling molecules called autoinducers globally regulate gene expression. Using quorum sensing, bacterial populations can switch from acting as individual cells to operating in a concerted, multi-cellular fashion [1]. In a clinical setting, a major challenge presented by biofilms is that bacteria living within them enjoy increased protection against host immune responses [2]–[8] and are markedly more tolerant to various anti-microbial treatments. A case in point is the opportunistic human pathogen *Pseudomonas aeruginosa*. *P. aeruginosa* is a Gram negative, biofilm-forming bacterium that has been shown to exhibit quorum sensing behavior using two distinct acyl-homoserine lactone (AHL) based pathways: the *rhlI/rhlR* pathway, which uses butyryl acyl homoserine lactone (C4-HSL), and the *lasI/lasR* pathway that uses 3-oxo-dodecanoyl homoserine lactone (3-oxo C12-HSL). A third signaling molecule, 2-heptyl-3-hydroxy-4(1 *H*)-quinolone, has been identified [9], and plays a role in *P. aeruginosa* virulence and possibly inter-species communication [10]. Multiple studies have shown that *P. aeruginosa* defective in QS may be compromised in their ability to form biofilms [11]–[14]. However, media composition and hydrodynamic conditions (independent of QS parameters) may also play a role in biofilm quality and stability [15]–[17].

One implication of the aforementioned observations is that the use of quorum sensing inhibitors (QSIs) may have the potential to circumvent the challenge of the development of antidrug resistance in the bacteria to which they are exposed. It is also conceivable that QSIs could be developed as an adjuvant to the administration of antibiotics, with the former serving to increase the susceptibility of infecting bacteria to cell death by exposure to antibiotics. Preliminary findings by Brackman *et al.* have demonstrated that QSIs increase the susceptibility of bacterial biofilms (including *P. aeruginosa* biofilms) to multiple types of antibiotics [18]. It has recently been demonstrated that some biofilm-associated bacteria return to the planktonic state through the secretion of D-amino acids [19] and *cis*-2-decenoic acid [20] natural products, both of which trigger biofilm disassembly. This finding supports the premise that small molecules that interrupt QS could serve as a means of controlling the establishment of bacterial infections. We report here the results of our work on the effects of various natural products and natural-products-inspired scaffolds as inhibitors of biofilm formation in *P. aeruginosa* and suggest a possible link to QSI. Further, we demonstrate that such compounds can reduce the bacterial load in the *Drosophila melanogaster* model of *P. aeruginosa* infection.

## Results and Discussion

### Effect of Compounds on Biofilms

**Figure 1:**
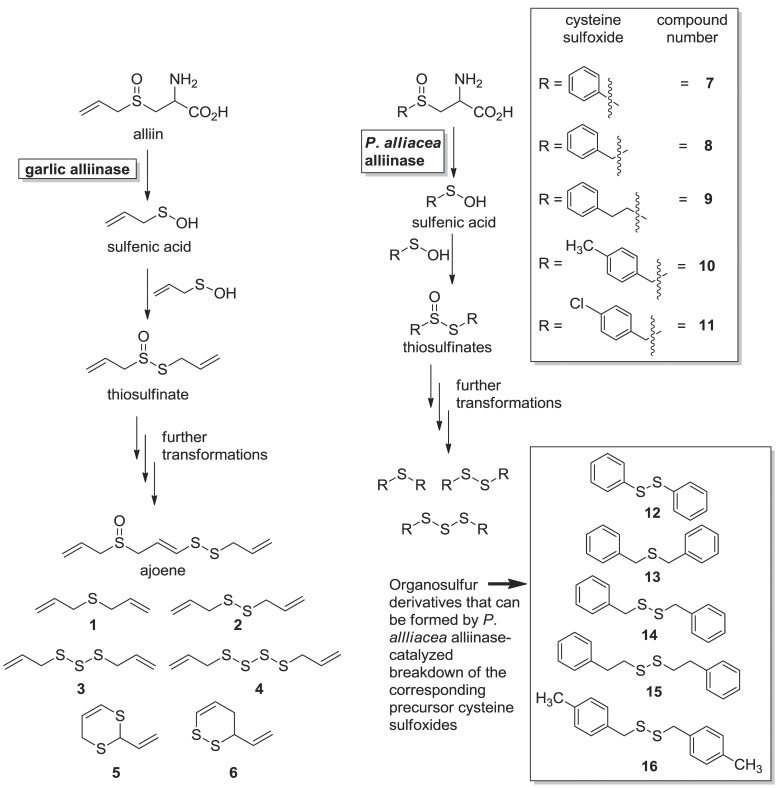
C-S lyase (i.e. alliinase) mediated cleavage of cysteine sulfoxides. For both the onion and *P. alliacea* alliinases, reaction with a cysteine sulfoxide derivative yields a fleeting sulfenic acid, two molecules of which can condense to give thiosulfinates. The thiosulfinates react further to yield a variety of organosulfur compounds. Compounds **1−6** from garlic have been found to inhibit *luxR*- and *lasR*-based QS systems. The *P. alliacea* alliinase has been shown to have broad substrate specificity and degrade a variety of cysteine sulfoxide derivatives such as compounds **7−11**.

Both *in vitro* and *in vivo* studies have shown the potential of garlic extracts to attenuate the virulence of *P. aeruginosa*. Harjai et al. [21] have observed that in a mouse model of nosocomial catheter-associated urinary tract infections, oral treatment with crude garlic extracts significantly lowered renal bacterial counts and protected mouse kidney from tissue destruction. The further observation of decreased production of virulence factors and reduced production of quorum-sensing signals by *P. aeruginosa* was interpreted to suggest that garlic exhibits QSI activity. Symth et al. observed a trend towards improvement in lung function in cystic fibrosis patients with garlic therapy. However, the sample size was too small to demonstrate statistically significant improvement in clinical outcomes [22]. Garlic derived natural products **1−6** (Figure 1) have been reported to inhibit *lasl/lasR*-based QS systems that are found in *Pseudomonas* species [23], as well as the *luxI/luxR* systems found in other bacteria, such as *Vibrio spp.* [24], [25]. Both *lux* and *las* QS systems utilize acyl homoserine lactone (AHL) compounds as autoinducers, although genetic regulation by these systems varies widely between different species. Bioassay guided fractionation of garlic extracts has revealed that the derivatives responsible for QSI activity include ajoene, as well as sulfides and polysulfides **1−4**, and vinyl dithiins **5** and **6** (Figure 1) [25]–[27]. Of these, **1−4** antagonized LuxR but were also toxic to bacteria. However, **5** and **6** possessed QSI activity exclusively in a LuxR monitor system [23]. All six of these compounds are derivatives of the cysteine sulfoxide alliin (Figure 1). These compounds are derivatives of a reaction cascade that begins in macerated garlic tissue with alliinase-catalyzed breakdown of the precursor *S*-alk(en)yl cysteine sulfoxide alliin (Figure 1). Similar alliinase-mediated chemistry occurs in the Amazonian medicinal plant *Petiveria alliacea* L. (Phytolaccaceae) to afford a variety of organosulfur derivatives with similar functionalities but different structures from those observed in garlic [28], [29]. A combination of natural and unnatural cysteine sulfoxides with which the *P. alliacea* alliinase reacts (**7−11**) [30] and several of the organosulfur compounds downstream of the action of its alliinase (**12−16**) [25], [28], [31], [32] are also shown in Figure 1. The demonstration that garlic-derived natural products inhibit *lux* and *las*-based QS systems [23] and the observation of the presence of the chemistry that produces these compounds in an increasing number of plants [28], [29], [31], [33], [34], implies that plants may have evolved to produce these secondary metabolites in order to serve as quorum sensing antagonists that prevent the establishment of infections by pathogenic microbes.

**Figure 2:**
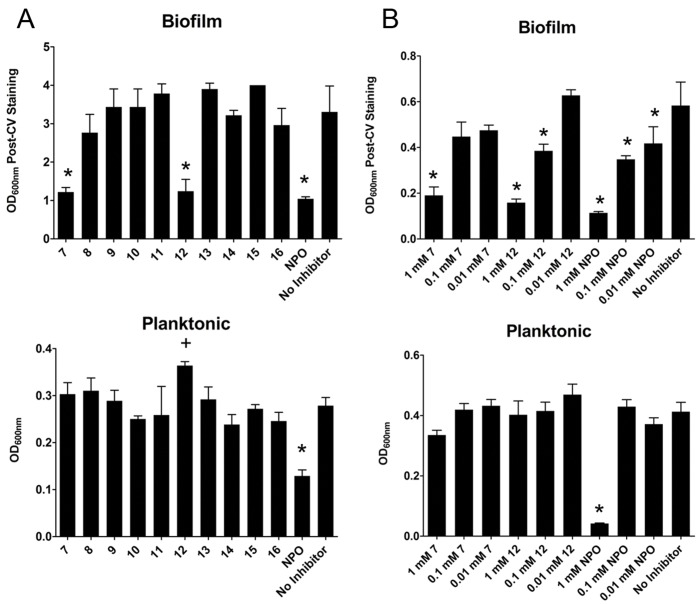
Panel A: Inhibition of *P. aeruginosa* PAO1 biofilm formation by small molecule inhibitors. Panel B: Concentration dependence for inhibition of *P. aeruginosa* PAO1 biofilm formation by small molecule inhibitors. For 2A and 2B, average OD_600nm_ measurements of crystal violet stained biofilms (top) and planktonic cells (bottom) are shown with error bars representing one standard deviation (n = 3). ANOVA (p<0.0001) was performed, followed by Tukey’s test, with asterisks (*) indicating significant (p<0.01) reduction in planktonic cell density or biofilm, and (+) representing a significant increase.

**Figure 3:**
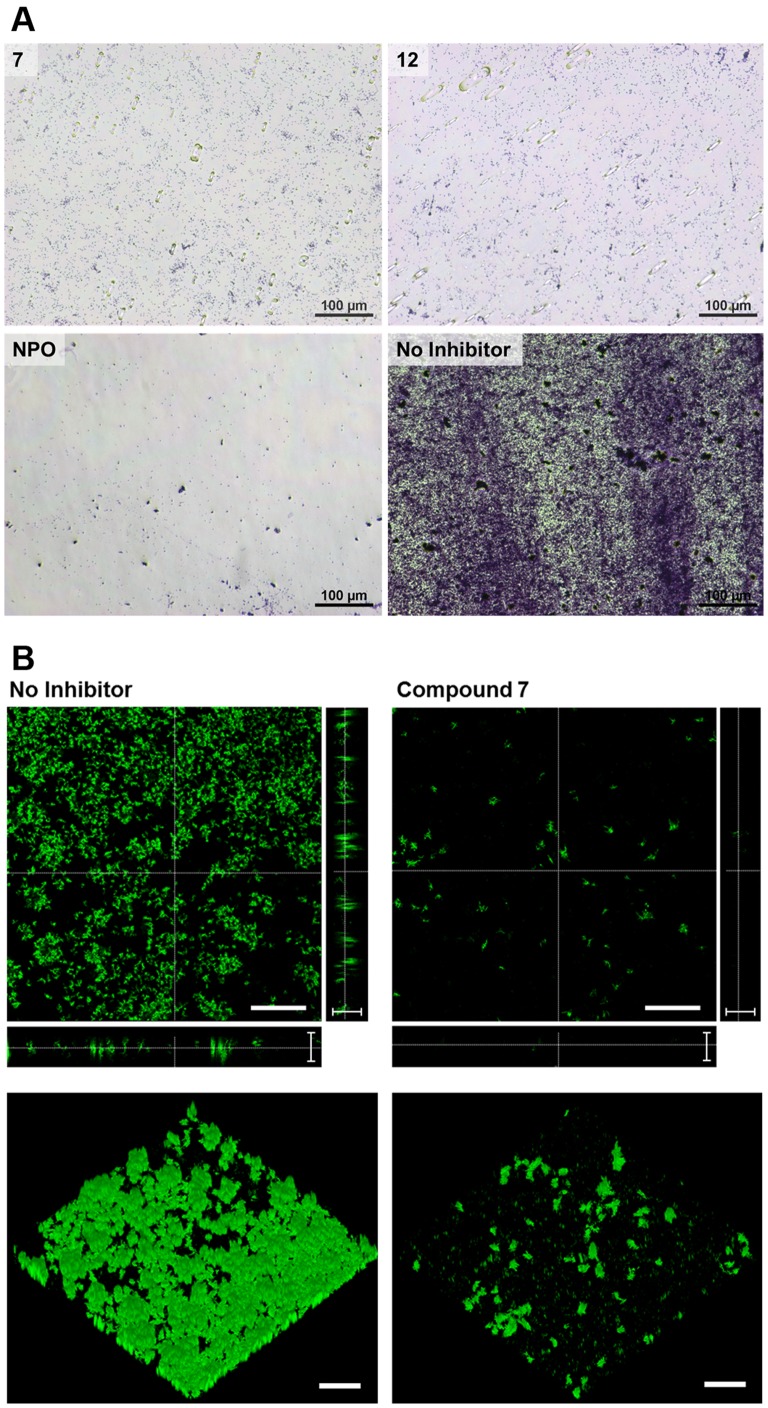
Panel A: Optical micrographs of bacterial biofilms. The films were grown in the presence of small molecule inhibitors **7** and **12** at 1 mM final concentration. Scale bar  = 100 µm. Panel B: Laser scanning confocal micrographs of biofilms. The films were grown in the presence of 1 mM compound **7** (right) or without inhibitor (left). Top down images are shown in the upper views, with vertical and horizontal cross-sections shown to the right and below, respectively. Three dimensional reconstructions are shown in the bottom views. Scale bars for top down and 3D views are 50 µm, while scale bars for cross sections are 10 µm.

To begin our investigations into this possibility, we determined the effect of five cysteine sulfoxides with which the *P. alliacea* alliinase has been shown to react (**7**−**11**, Figure 1) [30], as well as several of the sulfide and disulfide derivatives of the alliinase-mediated reactions (**12**−**16**, Figure 1), on biofilm formation and QS-based signaling in *P. aeruginosa*. The effects of these compounds on *P. aeruginosa* biofilm formation were initially assessed using a crystal violet-based biomass staining assay, with the results shown in Figure 2A. 4-Nitropyridine-*N*-oxide (NPO), a known inhibitor of biofilm formation in *P. aeruginosa*, was used as a positive control [32], [35]. Of the compounds tested, only *S*-phenyl-L-cysteine sulfoxide (**7)**, diphenyl disulfide (**12**) and NPO demonstrated significant biofilm inhibitory activity (Dunnett’s test, p<0.01), each at a concentration of 1 mM. These results were confirmed in optical micrographs of the resultant biofilms (Figure 3A), which show a significant difference in biofilm density on the bottom surface of the microplates in the presence of **7**, **12** and NPO as compared to the “no-inhibitor” control. Further, laser scanning confocal microscopy of a biofilm grown in the presence of compound **7** showed significantly altered 3D morphology, as compared to a no-inhibitor control (Figure 3B). In particular, the biofilm exposed to compound **7** is sparse and has limited 3D projections off of the glass substrate (Figure 3B, right), while the control biofilm (Figure 3B, left) is densely packed and extends vertically with multiple 3D projections.

**Figure 4:**
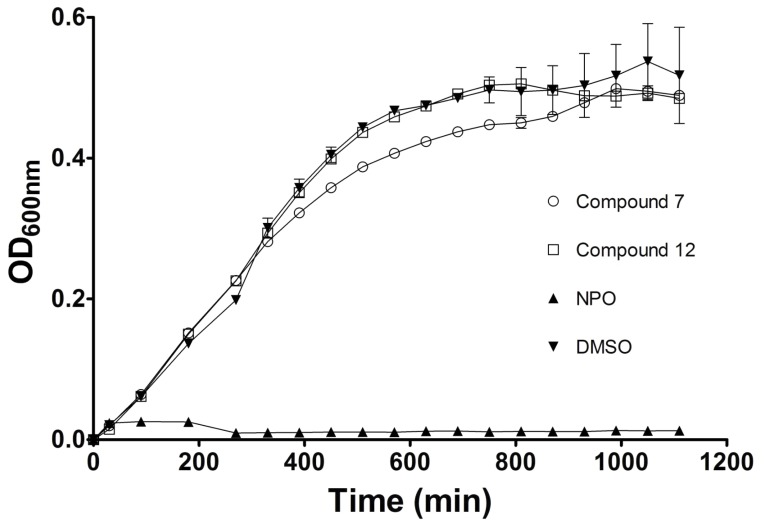
Growth curve analysis (OD_600nm_) for *P. aeruginosa.* The bacteria were grown in the presence of 1 mM of compounds **7**, **12**, NPO and DMSO. Error bars represent the standard deviation (n = 3).

Following this initial screening of compound effectiveness, compounds **7**, **12** and NPO were tested in a concentration dependent manner. Figure 2B shows the results of these experiments in which the concentrations of compounds **7**, **12** and NPO were varied from 0.01–1 mM. All three compounds exhibited significant (p<0.01) inhibitory activity against biofilm formation at 1 mM, but had diminished activity at lower concentrations. Of the three compounds, **12** retained significant inhibitory activity at 0.1 mM. To distinguish between the effects of **7**, **12** and NPO on cell growth vs. cellular biofilm forming ability, planktonic cell density was determined by measuring the OD of each microwell prior to biofilm staining with crystal violet. Our results show that, in addition to inhibiting biofilm formation, NPO significantly inhibits planktonic cell growth (Figure 2, panels A & B). In contrast, planktonic cell density was not significantly affected by compounds **7** and **12**. We further confirmed these results by performing growth curve analysis for compounds **7**, **12**, and NPO (Figure 4). *P. aeruginosa* cells exposed to 1 mM of compounds **7** and **12** showed no lag in growth, as compared to control cultures [containing only media and the solvent (DMSO) used for compound resuspension], and reached an optical density of >0.4 within 10 h. A parallel experiment using concentrations of up to 0.1 M of compounds **7** and **12** showed no difference in growth behavior (data not shown). In contrast, cells exposed to NPO showed no increase in OD, indicating complete inhibition of cell growth. These data suggest that NPO primarily affects biofilm formation by inhibiting bacterial growth, while compounds **7** and **12** affect biofilm formation independent of cell growth/propagation.

**Figure 5:**
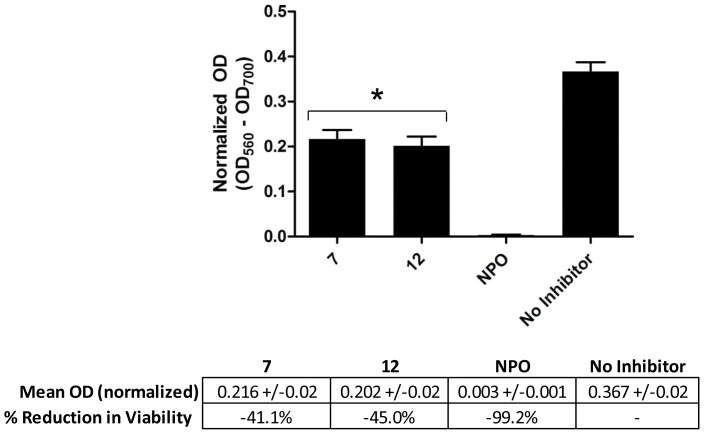
Cell viability within microplate-established biofilms as determined by the MTT assay. Biofilms were grown in the presence of 1 mM of compounds **7**, **12** and NPO. ANOVA (p<0.0001) was performed, followed by Tukey’s test, with asterisks (*) indicating significant (p<0.01) reduction in viability as compared to the no inhibitor control (n = 5).

To further evaluate the effects of the compounds on *P. aeruginosa* biofilms, the formazan-based 3-(4,5-dimethylthiazol-2-yl)-2,5-diphenyltetrazolium bromide (MTT) assay was performed on biofilms that were exposed to compounds **7**, **12** and NPO. This assay measures enzymatic activity in actively respiring cells and is therefore a measure of cell viability and/or relative numbers of viable cells. The analysis showed that biofilms grown in the presence of compounds **7** and **12** had significantly lower activity (41% and 45%, respectively) as compared to a no inhibitor control (Figure 5). In contrast, exposure to NPO reduced cell activity in the biofilm by >99%. These data further corroborate the results of the crystal violet-based staining assays, which showed a distinct decrease in stained biomass for biofilms grown in the presence of compounds **7**, **12** and NPO. The compounds did not however, inhibit planktonic cell growth, as demonstrated in the planktonic cell OD measurements shown in Figure 2, panels A & B, as well as the growth curve analysis shown in Figure 4. This is in stark contrast to the previously reported biofilm inhibitor NPO, which appears to inhibit both planktonic cell growth and biofilm formation. This distinction is important, since inhibition of planktonic growth and biofilm formation are decoupled for our compounds, and this inhibition does not rely upon strict biocidal activity.

### Effect of Compounds on Quorum Sensing

**Figure 6:**
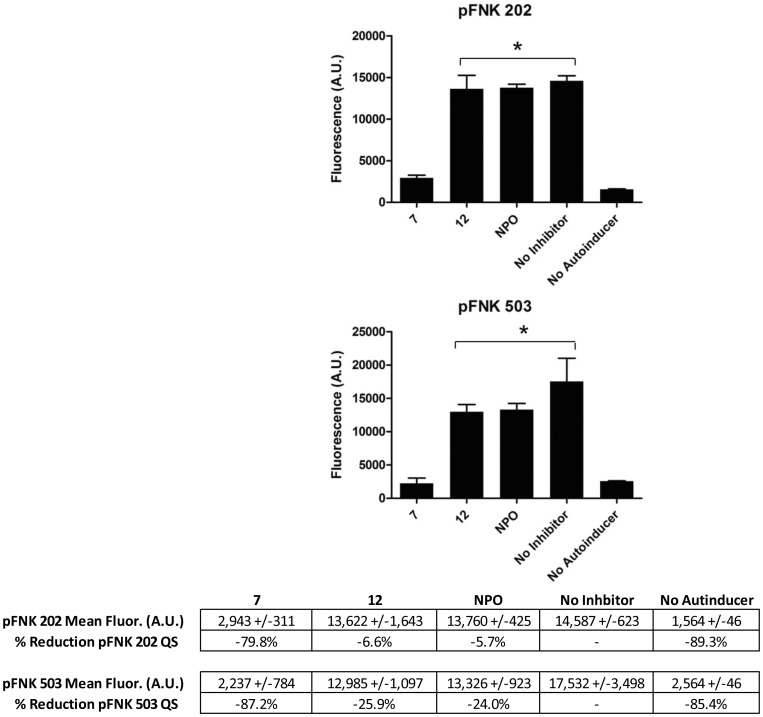
Inhibition of quorum sensing. In these experiments, *E. coli* pFNK202 (*rhlI/rhlR -* C4 HSL autoinducer) and pFNK503 (*lasI/lasR -* 3-oxo C12 autoinducer) reporters were used. Fluorescence intensity (480 nm/520 nm Ex/Em) of the resulting QS induced GFP expression was measured, following 24 h exposure to 1 mM of compounds **7, 12** and NPO. ANOVA (p<0.0001) was performed, followed by Tukey’s test, with asterisks (*) indicating significant (p<0.01) reduction in fluorescence as compared to the no inhibitor control (n = 3).

**Figure 7:**
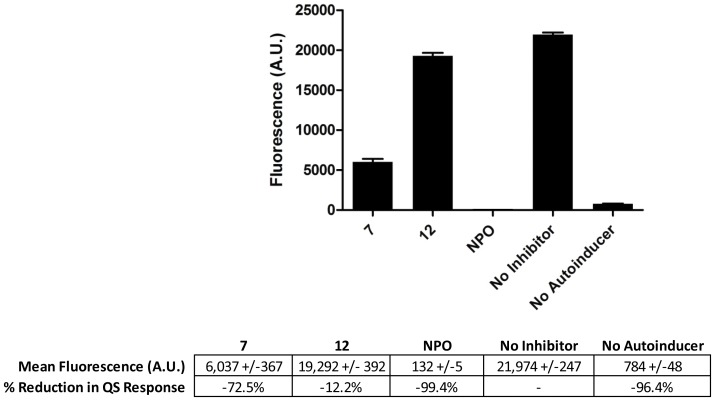
Inhibition of quorum sensing for the *P. aeruginosa* PAO-MWI pUM15 (lasI/lasR –3-oxo C12 autoinducer) reporter. Fluorescence intensity (480 nm/520 nm Ex/Em) of the resulting QS induced YFP expression was measured, following 24 h exposure to 1 mM of compounds **7**, **12** and NPO. ANOVA (p<0.0001) was performed, followed by Tukey’s pairwise comparison test, with asterisks (*) indicating significant (p<0.01) reduction in fluorescence as compared to the no-compound control (n = 3).

As described earlier, garlic-derived structural relatives of our active compounds demonstrated QSI activity in *lux*-based QS systems [23]. To determine if our active compounds behaved similarly, we evaluated their effects on QS using previously described quorum sensing reporter plasmids, pFNK202 and pFNK503 in *E. coli*, and in a *P. aeruginosa* reporter strain (PAO-MW1 with pUM15) [36]. The results of these experiments are shown in Figures 6 and 7. Compound **7** was shown to significantly affect the quorum sensing response (p<0.01), as measured by GFP expression, for both pFNK202 and pFNK503. This indicates that compound **7** can antagonize both the *lasI/lasR* and *rhlI/rhlR* quorum sensing systems in this artificial reporter strain. Compound **7** also significantly (p<0.01) affected quorum sensing in the *P. aeruginosa* PAO-MW1 pUM15 reporter, further supporting the conclusion that it is operating through antagonism of the *lasI/R* quorum sensing system. To further examine the effect of compound **7** on quorum sensing, we used the PAO-MW1 reporter containing pUM15 in our biofilm inhibition assay. The reporter itself is deficient in both *rhlI* and *lasI* (*rhlI*::Tn*501 lasI*::*tetA*) which makes it a QS mutant when exogenous autoinducer is not provided. We exposed this strain to both exogenous autoinducer (3-oxo C12 HSL) alone, and to 3-oxo C12 HSL plus compound **7**, and evaluated biofilm formation. This experiment showed that exposure to compound **7** reduced biofilm formation by >50% (data not shown). When no autoinducer was added (completely shutting off quorum sensing), biofilm formation by both the control (no compound added) and compound **7** exposed cells was reduced by 80% (data not shown). Further, there was no significant difference between biofilm formation in these two exposure groups. This follows previous studies in which elimination of quorum sensing significantly affected biofilm formation in *P. aeruginosa* [11]–[14]. We conclude that complete inhibition of quorum sensing masks the effect of compound **7** on biofilm formation and cannot distinguish between QSI or another mechanism responsible for biofilm inhibition.

Unlike compound **7**, compound **12** did not significantly affect the quorum sensing response for the *E. coli* reporters pFNK202 or pFNK503, but did show significant antagonism in the *P. aeruginosa* reporter (12% reduction in fluorescence). None of the other compounds significantly affected the quorum sensing response (data not shown). The control inhibitor, NPO, did not show an effect on quorum sensing in either of the *E. coli* reporters, but did reduce quorum sensing in the *P. aeruginosa* reporter by 99%. This is likely due to the difference between the assays used for *E. coli* vs. *P. aeruginosa,* since the *P. aeruginosa* assay was dependent upon cell growth and expression of the fluorescent reporter protein. As shown previously, NPO inhibits growth of *P. aeruginosa,* which would likewise prevent expression of the reporter (YFP) in this assay. Since both compounds **7** and **12** were shown to inhibit biofilm formation, but do not have similar QSI activity, they may be functioning via different pathways, or may be additionally processed by the cells to yield alternative compounds or breakdown products.

### Effect of Compounds on Infection in D. melanogaster

**Figure 8:**
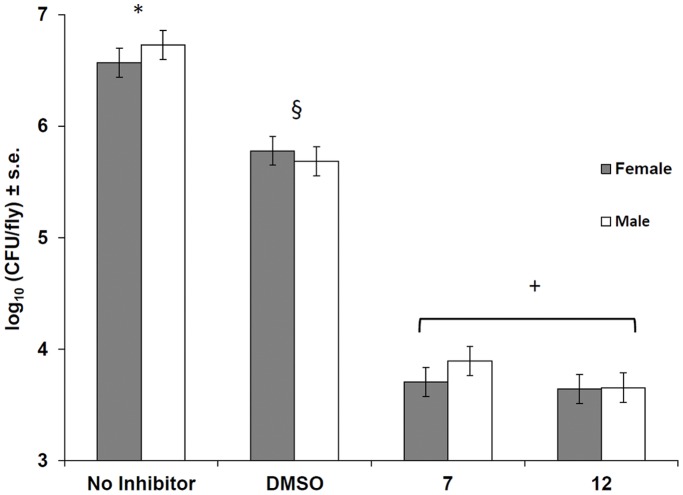
Effects of compounds 7 and 12 on *D. melanogaster* infected with *P. aeruginosa* PAO1. Treatment groups not connected by the same symbol are significantly different from each other, as determined by ANOVA (p<0.01) and Tukey’s test (p<0.05).

It has been observed that biofilm formation can have profound effects on infection dynamics and pathogenesis [37]. Recently, the model organism *Drosophila melanogaster* has been used to study *in vivo P. aeruginosa* infection dynamics, and in particular, the relationship between biofilm formation and pathogenicity [38], [39]. Thus, Estin *et al*. have shown that *P. aeruginosa* infection and lethality in *Drosophila* is QS-dependent and that expression of a human-derived autoinducer degrading enzyme (paraoxonase 1) can act as a protective agent to reduce infection [38]. Fly mortality (reflecting pathogen virulence) is a composite measure of host defense incorporating both resistance and tolerance to the infection. Previous experiments in our labs (using *Pseudomonas aeruginosa* infections) have shown a strong positive correlation between CFU counts and fly mortality (data not shown). In this study, we used the *Drosophila* infection model to assess the effect of compounds on patterns of resistance (which is defined as the inverse of bacterial load). We observed a dramatic decrease in the number of bacteria recovered from flies treated with either compound **7** or **12** compared to controls (Figure 8). Both compounds had similar effects on slowing bacterial growth in flies. The solvent used to dissolve the compounds (DMSO) appears to slow bacterial growth during infection, but to a much lesser extent than either compound **7** or **12** (Figure 8). Similar effects were seen for both male and female flies, and therefore no sex bias was observed. For these experiments, bacteria were suspended in a 0.1 M concentration of compound that was then injected into the flies. Bacteria were only exposed to this high concentration for 5 min or less, since injected material is expected to be diluted inside of the flies. Further, 0.1 M concentrations of compounds **7** and **12** were not shown to affect *P. aeruginosa* cell growth dynamics (as determined by growth curve analysis). Therefore the reduced bacterial load in affected flies is attributed to increased ability of the flies to mitigate infection, and not compound induced reduction in bacterial growth rate.

Our results indicate that the disruption of biofilm formation (or QS) may significantly alter infection dynamics in acute systemic infections in *D. melanogaster.* We propose that the inability to form a biofilm during acute systemic infections may make the growing bacterial population more susceptible to the innate immune defense of the fly. Our results seem in contrast to those of a recent study that found that strains of *P. aeruginosa* not capable of producing a biofilm showed increased virulence in the fly [39]. However, in that study, flies were fed bacteria that resulted in chronic infections maintained largely in the crop. Further, strains capable of biofilm formation were better inducers of local antimicrobial peptide production in the crop, indicating that the biofilm is more immunogenic than planktonic cells [39]. In acute systemic infections such as those carried out here, innate immune induction due to both wounding and the recognition of the bacteria are likely to occur, and the inability to form a biofilm may leave the population more prone to attack by these defenses.

The structural relationship between the two active compounds **7** and **12** is noteworthy because of the clues it provides on how the active compounds may be metabolized in *P. aeruginosa*. When a C-S lyase such as the *P. alliacea* alliinase encounters **7** or another cysteine sulfoxide of appropriate structure, the C−S( = O) bond that is β- to the amino acid moiety is cleaved to yield a sulfenic acid, two molecules of which rapidly condense to yield a thiosulfinate (Figure 1). The labile thiosulfinates can then undergo further transformations to yield disulfides. In the case of **7**, the corresponding disulfide is **12**. C-S lyase mediated decompositions of cysteine sulfoxides are marked by the emergence of a strong sulfurous odor as the disulfide is generated. The smell is characteristic and easily detectable since the precursor cysteine sulfoxides are non-volatile compounds. Over the course of our experiments, we observed that with time, the *P. aeruginosa* samples that were incubated with cysteine sulfoxides **7**−**11** all began to emit an organosulfur odor reminiscent of fresh *P. alliacea* macerates. The implication of this observation is that the bacteria possess a C-S lyase enzyme analogous to those observed in garlic and *P. alliacea*, that can degrade cysteine sulfoxides to ultimately yield sulfides and/or disulfides that might themselves exhibit biofilm inhibitory activity. Although there are no literature reports that show the PAO1 strain of *P. aeruginosa* to possess cysteine sulfoxide lyases, a cysteine sulfoxide lyase has been isolated from *Pseudomonas cruciviae* [40]. An amino acid BLAST search [41] of the *P. aeruginosa* PAO1 genome yields a hypothetical protein sequence with 26% sequence similarity (and total alignment score of 31.6) to the *Allium cepa* (onion) C-S lyase (GenBank: AF126049.1). The sequence similarity is relatively low; however, this putative protein could indeed have C-S lyase enzymatic activity. This possibility would need to be demonstrated or confirmed in a follow-on study. A further implication of our findings is that the cysteine sulfoxides enter into the bacterial cells, since their decomposition requires the action of lyase enzymes that would not likely be present in the extracellular milieu, but rather in the cytosol.

Since it has been demonstrated that structurally similar garlic-derived organosulfur compounds can inhibit *lux*-based bacterial quorum sensing [23] and that compound **7** identified in this study inhibits *las* and *rhl-*based quorum sensing, we hypothesize that its inhibition of biofilm formation in *P. aeruginosa* may occur through antagonism of quorum sensing pathways. The fact that compound **12** did not show significant quorum sensing inhibition, but did inhibit biofilm formation, suggests that it is functioning via a different mechanism, or its QSI activity is obscured by the particular QSI assays that were used. Nevertheless, our results reveal a new class of bacterial biofilm inhibitors, and further support an approach to biofilm inhibition via antagonism of quorum sensing behavior. Further, we have demonstrated that these compounds can significantly affect the bacterial load in a *Drosophila* based infection model, which suggests their applicability for mitigating *P. aeruginosa* infections. Studies are underway to elucidate the specific mechanism of action of these compounds in biofilm inhibition, to identify their mode of action during *Drosophila* infection, and to further characterize their QSI activity.

## Materials and Methods

### Instrumentation

NMR spectra were recorded on a Bruker 400 MHz spectrometer. IR spectra were recorded using a Perkin Elmer Spectrum 100 FT-IR spectrophotometer.

### Compounds

The *S*-phenyl- and *S*-benzyl-L-cysteines (precursors to compounds **7** and **8** respectively), as well as diphenyl disulfide, dibenzyl sulfide and dibenzyl disulfide (compounds **12**, **13**, and **14** respectively) were purchased from Sigma-Aldrich (St. Louis, MO, USA) and were used without further purification. Compound **8** was synthesized as previously described [29]. *S*-β-Phenylethyl-, *S*-[(4-methylphenyl)methyl]- and *S*-[(4-chlorophenyl)methyl]-L-cysteines (precursors to compounds **9**, **10**, and **11** respectively) were synthesized as described by Kubec and Musah [29]. Briefly, to a stirred solution of L-cysteine (0.1 mol) in 150 ml of 1 M NaOH and 200 ml of ethanol was added the corresponding bromide (1.15 equiv). After 2 h, the solution was acidified with conc. HCOOH to pH 5–6. The precipitated product was filtered off and washed thoroughly with acetone and diethyl ether. Cysteine sulfoxides **7** and **9**–**11** were prepared by oxidation of the corresponding *S*-substituted L-cysteines with H_2_O_2_. Bis(2-phenylethyl)disulfide and bis[(4-methylphenyl)methyl] disulfide (compounds **15** and **16** respectively) were synthesized by NaIO_4_ oxidation of the corresponding thiols as described by Montazerozohori *et al*. [42]. Briefly, 0.5 equivalents of NaIO_4_ was placed in a mortar and moistened with water. Then 1 equivalent of the thiol was added and the mixture was triturated for 2 min. The solid material was taken up in CH_2_Cl_2_ and the resulting solution filtered. The filtrate was dried over MgSO_4_ and the solvent was evaporated under reduced pressure to yield the disulfide product.

***S*** **-**(**β-Phenylethyl**)**-L-cysteine (precursor to compound 9):** white solid; mp 220−221°C; IR ν_max_ cm^−1^: 2675−3150 (*m, br*), 1680 (*m, sh*), 1577 (*m, sh*), 1479 (*m, sh*), 1408 (*m, sh*), 695 (*s, sh*); ^1^H NMR (400 MHz; D_2_O/NaOD; DSS): *δ* 2.76 (1H, *dd*, *J* = 8.0, 14.8 Hz, H-3a), 2.84−2.94 (5H, *m*), 4.38 (1H, *dd*, *J* = 2.4, 8.0 Hz, H-2), 7.36−7.42 (5H, *m*, H_arom_); ^13^C NMR (400 MHz; D_2_O/NaOD; DSS): *δ* 33.0, 36.8, 37.4, 55.0, 126.4, 128.5, 128.6, 140.6, 180.0; HR ESI-TOF [MH^+^]: 226.0890 (calc. for C_11_H_15_NO_2_S: 226.0896).

***S*** **-[(4-Methylphenyl)methyl]-L-cysteine (precursor to compound 10):** white solid; mp 222−223°C (boiling water); IR ν_max_ cm^−1^: 2510−3220 (*m, br*), 1616 (*m, sh*),1584 (*s, sh*), 1558 (*s, sh*), 1492 (*s, sh*), 1393 (*s, sh*), 821 (*m, sh*); ^1^H NMR (400 MHz; D_2_O/NaOD; DSS ): 2.31 (3H, s), 2.69 (1H, *dd*, *J* = 8.4, 12 Hz, H-3a), 2.76 (1H, *dd*, *J* = 2, 12 Hz, H-3b), 3.38 (1H, *dd*, 4.0, 12.0 Hz, H-2), 3.74 (2H, s, H-5), 7.21−7.29 (4H, *m*, H_arom_); ^13^C NMR (400 MHz; D_2_O/NaOD; DSS): *δ* 20.0, 35.1, 36.2, 54.8, 128.8, 129.2, 135.3, 137.3, 181.0; HR ESI-TOF [MH^+^]: 226.0900 (calc. for C_11_H_15_NO_2_S: 226.0896).

***S*** **-[(4-Chlorophenyl)methyl]-L-cysteine (precursor to compound 11):** white solid; mp 207–209°C (boiling water); IR ν_max_ cm^−1^: 2625−3215 (*m, br*), 1618 (*m*), 1588 (*m, sh*), 1562 (*m, sh*), 1490 (*s, sh*), 1394 (*m, sh*), 1094 (*m, sh*), 839 (*m, sh*), 730 (*m, sh*); ^1^H NMR (400 MHz; D_2_O/NaOD; DSS): *δ* 2.76 (1H, *dd*, 8, 12 Hz, H-3a), 2.69 (1H, *dd*, 4.0, 12.0 Hz, H-3b), 3.37 (1H, *dd*, 6.4, 12.0 Hz, H-2), 3.66 (2H, s, H-5), 7.34−7.39 (4H, *m*, H_arom_); ^13^C NMR (400 MHz; D_2_O/NaOD; DSS): *δ* 34.7, 36.1, 54.8, 128.5, 130.3, 132.2, 137.1, 181.0; HR ESI-TOF [MH^+^]: 246.0353 (calc. for C_10_H_12_ClNO_2_S: 246.0350).

***S*** **-Phenyl-L-cysteine sulfoxide** **(7):** white solid; mp 158−160°C (boiling water); IR ν_max_ cm^−1^: 2476−3165 (*m, br*), 1583 (*s*), 1516 (*m*), 1401 (*m*), 1032 (*s, sh*), 746 (*m, sh*), 688 (*s, sh*); ^13^C NMR (400 MHz; D_2_O, NaOD): 38.8, 54.9, 126.5, 129.1, 129.5, 134.5, 180.5; HR ESI-TOF [MH^+^]: 214.0533 (calc. for C_9_H_11_NO_3_S: 214.0532).

***S*** **-**(**β-Phenylethyl**)**-L-cysteine sulfoxide** **(9):** white solid; mp 179−180°C; IR ν_max_ cm^−1^: 2500−3175 (*m, br*), 1651 (*m*), 1582 (*s, sh*), 1399 (*m*, *sh*), 1320 (*m, sh*), 1023 (*s, sh*), 700 (*s, sh*); ^13^C NMR (400 MHz; D_2_O/NaOD; DSS): *δ* 52.0, 52.6, 56.5, 56.7, 126.9, 128.6, 128.8, 138.7, 179.3; HR ESI-TOF [MH^+^] 242.0850 (calc. for C_11_H_15_NO_3_S: 242.0845).

***S*** **-[(4-Methylphenyl)methyl]-L-cysteine sulfoxide** (**10):** white solid; mp 169−171°C; IR ν_max_ cm^−1^: 2530−3170 (*m*, *br*), 1583 (*s*),1514 (*m*, *sh*), 1420 (*m*, *sh*), 1356 (*m*, *sh*), 1016 (*s*, *sh*), 817 (*m, sh*); ^13^C NMR (400 MHz; D_2_O/NaOD; DSS): *δ* 20.22, 50.88, 55.59, 125.93, 129.49, 130.34, 138.93, 179.79; HR ESI-TOF [MH^+^]: 242.0842 (calc. for C_11_H_15_NO_3_S: 242.0845).

***S*** **-[(4-Chlorophenyl)methyl]-L-cysteine sulfoxide** (**11):** white solid; mp 170−172°C; IR ν_max_ cm^−1^: 2521−3165 (*m, br*), 1577 (*m, br*), 1492 (*m, sh*), 1388 (*m, sh*), 1019 (*s, sh*), 824 (*m*); ^13^C NMR (400 MHz; D_2_O, NaOD): *δ* 51.0, 55.6, 55.7, 127.8, 128.8, 131.8, 134.0, 179,2; HR ESI-TOF [MH^+^]: 262.0303 (calc. for C_10_H_12_ClNO_3_S: 262.0299).

**Bis(2-phenylethyl) disulfide** (**15):** viscous pale yellow oil; IR ν_max_ cm^−1^: 3026 (*w, sh*), 2911 (*w*), 1603 (*w, sh*), 1495 (*m, sh*), 1453 (*m, sh*), 748 (*m*); ^1^H NMR (CDCl_3_, TMS): *δ* 2.91–3.01 (4H, m), 7.18–7.31 (5H, H_arom_); ^13^C NMR (400 MHz; CDCl_3_; TMS): *δ* 35.8, 40.2, 126.5, 128.6, 128.7, 140.1; HR ESI-TOF [MH^+^]: 275.0922 (calc. for C_16_H_18_S_2_: 275.0923).

**Bis[(4-methylphenyl)methyl] disulfide** (**16):** white solid; mp 42−44°C; IR ν_max_ cm^−1^: 3019 (*w, sh*), 2914(*w, sh*), 1510 (*m, sh*), 1101 (*m, sh*), 814 (*s, sh*); ^1^H NMR (400 MHz; CDCl_3_, 0.03% TMS): *δ* 2.33 (*s*, 3 H), 3.61 (*s*, 2 H), 7.12 (4H, H_arom_); ^13^C (400 MHz; CDCl_3_; TMS): *δ* 21.3, 43.3, 129.3, 129.4, 134.5, 137.2; HR ESI-TOF [MH^+^]: 275.0918 (calc. for C_16_H_18_S_2_: 275.0923).

### Biofilm Inhibition

*P. aeruginosa* PAO1 was propagated on trypticase soy agar (TSA) for plate-based assays or in trypticase soy broth (TSB) for liquid culture. M9 growth media supplemented with 0.4% (w/v) glucose and 0.4% (wt/v) casamino acids was used for biofilm formation experiments. Culture media (TSB, TSA, M9 salts and casamino acids) were obtained from Difco/Becton Dickinson (Franklin Lakes, NJ, USA) and all other reagents (phosphate buffered saline, glucose, ethanol and crystal violet) were obtained from Sigma-Aldrich (St. Louis, MO, USA). Corning 35–1172 flat-bottomed polystyrene 96-well plates were used for biofilm formation experiments and optical density measurements were performed in a Tecan M-200 (Durham, NC, USA) plate reader. Optical micrographs of biofilms were obtained using a Nikon Eclipse 80i microscope.

A microplate based assay, modified from Junker *et al.* [32] was used to screen compounds for QSI. Briefly, *P. aeruginosa* PAO1 was grown in TSB for 18 h at 37°C with rotary shaking at 225 rpm. The culture was then centrifuged at 14,000 rpm and rinsed with phosphate buffered saline (PBS, pH 7.4) three times, then was re-suspended in M9 minimal growth media to approximately 1×10^7^ cfu/ml (determined by OD and plate count assay). Test compounds were dissolved in DMSO and were added to sterile distilled water to achieve concentrations ranging from 0.1–10 mM while keeping DMSO at a maximum of 1% (v/v). *P. aeruginosa* inocula (360 µl) were then pre-mixed with 40 µl of the test compound solutions to achieve final compound concentrations ranging from 0.01–1 mM. An aliquot (100 µl) of this cell/compound mixture was then added to three separate wells in a 96-well microplate for replicate testing. For control wells (no inhibitor), dilute DMSO was added to the inocula instead of test compounds, to a final concentration of 1% (v/v). Optical density (OD_600nm_) measurements were performed immediately after inoculation and after 24 h incubation at 37°C (without shaking) to monitor planktonic cell growth. To determine the amount of biofilm formation, supernatant from the microplate wells was gently removed and the wells were washed twice with 150 µl of PBS using a multichannel pipette. The remaining biofilm was then stained using 100 µl of a 0.2% (w/v) crystal violet solution for 15 min at room temperature. The crystal violet was then removed from the wells, the wells were rinsed four times with PBS, and then 100 µl of 95% ethanol was added to extract the crystal violet solution from the biofilm. The OD_600nm_ of the extracted crystal violet was then measured, yielding a measure of biofilm formation (relative to the control). For optical imaging, crystal violet stained biofilms were washed with distilled water and no ethanol extraction was performed.

In addition to crystal violet based quantification of biofilm biomass, cell viability within biofilms exposed to inhibitor compounds was determined using the formazan dye-based MTT assay (Cell Proliferation Kit I, Roche Diagnostics, Mannheim, Germany). This assay has previously been described for determination of biofilm cell viability [43]–[45]. Briefly, biofilms were grown in 96 well microplates for 24 h as described above, in the presence and absence of inhibitor compounds. After this initial inoculation period, planktonic cells were removed and the remaining biofilm was gently rinsed three times with 100 µl of PBS. After rinsing, 100 µl of PBS and 10 µl of the MTT labeling reagent were added and the suspension was incubated for 4 h at 37°C, followed by addition of 100 µl of solubilization solution. Plates were then incubated for 24 h at 37°C and absorbance measurements were taken using a Tecan M-200 plate reader at 560 nm (peak absorbance for the formazan dye breakdown product) and at 700 nm (reference wavelength for the intact dye).

### Confocal Imaging

To prepare biofilms for confocal imaging, cells were cultured at 37°C overnight in TSB. Biofilms were grown on a 50 mm glass bottom dish (Willco Wells B.V., Amsterdam, The Netherlands) by diluting the overnight cell culture to 1% in 10% TSB in filter-sterilized deionized water. Compounds were added to the experimental samples to a final concentration of 1 mM, and filter-sterilized deionized water was used in place of compounds in the control samples. Samples were incubated without shaking for 48 h at 37°C. Biofilms were then stained with FilmTracer FM 1–43 fluorescent biofilm cell stain (Invitrogen) at a final concentration of 1 µg/ml. Imaging was performed with a Leica TCS SP5 II confocal microscope, using a 20X oil-immersion lens with 477 nm excitation and 560–600 nm emission range. Both image acquisition and subsequent manipulation were performed using Leica Application Suite v2.1.2 software.

### Quorum Sensing Inhibition

Quorum sensing inhibition (QSI) studies were performed in both *Escherichia coli* and *P. aeruginosa* biosensor strains which respond to exogenously added autoinducers by expressing fluorescent proteins (GFP or YFP). Experiments using *E. coli* were performed using quorum sensing reporter plasmids pFNK-503-qscrsaL (abbreviated pFNK503) or pFNK-202-qsc119 (abbreviated pFNK202) in *E. coli* JM2.300, as previously reported by Brenner *et al.* [46]. These plasmids were kindly provided by Dr. Ron Weiss (Massachusetts Institute of Technology, Cambridge, MA, USA). Plasmid pFNK503 contains part of the *P. aeruginosa lasI/R* pathway, including the *lasR* gene and the green fluorescent protein (*gfp*) gene under the control of the *pLas* promoter. Cells hosting this plasmid respond to 3-oxo C12-HSL by producing GFP. Plasmid pFNK202 contains part of the *P. aeruginosa rhlI/R* pathway, including the *rhlR* gene and the green fluorescent protein (*gfp*) gene under the control of the pRhl promoter. Cells hosting this plasmid respond to C4-HSL by producing GFP. Both strains pFNK503 and pFNK202 were maintained on trypticase soy agar (TSA) plates containing 0.5 µg/ml kanamycin (Kan) at 37°C. QSI studies in *P. aeruginosa* were performed using a quorum sensing reporter strain previously described by Muh *et al.* [36]. This reporter strain, obtained from Dr. Peter Greenberg (University of Washington, Washington State, USA) uses pUM15 (*rasL*::*yfp* transcriptional fusion, Cb^r^) in a *P. aeruginosa* PAO-MW1 (*rhlI*::Tn*501 lasI*::*tetA*) background. This reporter expresses YFP when exposed to 3-oxo C12-HSL at concentrations as low as 0.3 µM [36]. Cultures were maintained on Luria-Bertani Broth (LB) plates containing 150 µg/ml carbenicillin at 37°C.

Liquid cultures of *E. coli* reporter strains were grown in TSB +0.5 µg/ml Kan at 37°C with shaking at 225 rpm. For QSI experiments, strains were grown for 12 h to an OD_600_ of 0.7. Aliquots of cells (80 µl) were then added to individual wells of a sterile 96 well microplate. Autoinducers (10 µl of 1 mM 3-oxo C12-HSL or C4-HSL, Cayman Chemicals, Ann Arbor, MI) were then added to each well. This addition was followed by addition of test compounds (10 µl of a 10 mM stock solution) to yield a final concentration of 1 mM, prepared as described above. Control wells contained TSB only, cells without autoinducer, cells with autoinducer but no test compound, or cells with autoinducer and 1 mM DMSO (to determine if the DMSO component of the test compound stocks affected quorum sensing response). *P. aeruginosa* QSI experiments were performed using a similar protocol with the following modifications. The *P. aeruginosa* reporter was grown in LB with 150 µg/ml carbenicillin, and 10 µl of a mid-log phase culture was added per microwell. Microwells were brought to 100 µl total volume with sterile LB and the autoinducer 3-oxo C12-HSL was added to a final concentration of 0.1 mM. For both *E. coli* and *P. aeruginosa* reporters, an initial fluorescence reading (480 nm excitation/520 nm emission) was then taken for each well (in a Tecan M200 microplate reader), followed by incubation at 37°C without shaking. Fluorescence readings were then repeated at 24 h to evaluate the amount of GFP expression for each experimental condition. The percent change in fluorescence intensity was determined for each test condition and a minimum of three replicate samples was used for all experiments.

### Effect of Compounds on Infections in Drosophila Melanogaster

In recent years *D. melanogaster* has emerged as a powerful model system for understanding *P. aeruginosa* pathogenicity [39], [47]. Thus, the effects of compounds on *in vitro* infections were tested using *D. melanogaster* infected with *P. aeruginosa* strain PAO1. For infections, cells were grown to log-phase in LB broth at 37°C in a shaking incubator. Cultures were diluted using sterile LB to a concentration of 2×10^7^ cfu/ml for injections. There were three treatment groups: (i) cells alone; (ii) cells with DMSO (10% v/v final concentration); and (iii) cells and 0.1 M compounds. Stock solutions of compounds were made in DMSO. Growth curve analysis, as described above, was performed using 0.1 M concentrations of compounds to demonstrate *P. aeruginosa* viability at elevated compound concentrations. A volume of 54 nl for each treatment culture was injected into the thorax of flies using a Nanoject II nanoliter injector (Drummond Scientific), corresponding to ∼1000 cfu per fly. A total of 16 males and 16 females were injected for each treatment group. Single flies were homogenized 18 h post injection in 250 µl of sterile LB, diluted to 1% of its original concentration. An aliquot of this sample (50 µl) was plated using an Autoplate 4000 spiral plater (Spiral Biotech, Bethesda, MD, USA). Plates were incubated overnight at 30°C and the number of colony forming units (cfu) was counted using the Q-Count detection system (Spiral Biotech, Bethesda, MD, USA). Colony counts were natural log transformed prior to analysis using analysis of variance (ANOVA). The statistical model included the main effects of treatment and sex as well as the combined treatment/sex interaction.

### Statistics

Statistically significant variance (p<0.01) for collected data was determined by ANOVA. Data with significant variance were further analyzed by Dunnett’s multiple comparison test or Tukey’s Honestly Significant Difference (HSD) test. For fly infection experiments, pair-wise comparison of the different treatment groups was carried out using Tukey’s HSD test with an experiment-wise error rate of α ≤0.05. Statistics were performed using GraphPad Prism 5 software (Graph Pad Software Inc., La Jolla, CA, USA).

## References

[1] Kaufmann GF, Park J, Janda KD (2008). Bacterial quorum sensing: a new target for anti-infective immunotherapy.. Expert Opin Biol Ther.

[2] Dasgupta MK (1996). Biofilm causes decreased production of interferon-gamma.. J Am Soc Nephrol.

[3] Meluleni GJ, Grout M, Evans DJ, Pier GB (1995). Mucoid *Pseudomonas aeruginosa* growing in a biofilm *in vitro* are killed by opsonic antibodies in the mucoid exopolysaccharide capsule but not by antibodies produced during chronic lung infection in cystic fibrosis patients.. J Immunol.

[4] Costerton JW, Lewandowski Z, Caldwell DE, Korber DR, Lappin-Scott HM (1995). Microbial biofilms.. Annu Rev Microbiol.

[5] Rogers FP J, Orliff C (1994). The effects of extracellular slime from *Staphylococcus epidermis* on phagocytic ingestion and killing.. FEMS Immunol Med Microbiol.

[6] Jensen ET, Kharazmi A, Garred P, Kronborg G, Fomsgaard A (1993). Complement activation by P*seudomonas aeruginosa* biofilms.. Microb Pathog.

[7] Costerton JW, Stewart PS, Greenberg EP (1999). Bacterial biofilms: a common cause of persistent infections.. Science.

[8] Davies D (2003). Understanding biofilm resistance to antibacterial agents.. Nat Rev Drug Discov.

[9] Pesci EC, Milbank JB, Pearson JP, McKnight S, Kende AS (1999). Quinolone signaling in the cell-to-cell communication system of *Pseudomonas aeruginosa*.. Proc Natl Acad Sci U S A.

[10] Reen FJ, Mooij MJ, Holcombe LJ, McSweeney CM, McGlacken GP, et al. The *Pseudomonas* quinolone signal (PQS), and its precursor HHQ, modulate interspecies and interkingdom behaviour.. FEMS Microbiol Ecol.

[11] De Kievit TR, Gillis R, Marx S, Brown C, Iglewski BH (2001). Quorum-sensing genes in *Pseudomonas aeruginosa* biofilms: their role and expression patterns.. Appl Environ Microbiol.

[12] Sakuragi Y, Kolter R (2007). Quorum-sensing regulation of the biofilm matrix genes (pel) of *Pseudomonas aeruginosa*.. J Bacteriol.

[13] Davies DG, Parsek MR, Pearson JP, Iglewski BH, Costerton JW (1998). The involvement of cell-to-cell signals in the development of a bacterial biofilm.. Science.

[14] Hentzer M, Riedel K, Rasmussen TB, Heydorn A, Andersen JB (2002). Inhibition of quorum sensing in *Pseudomonas aeruginosa* biofilm bacteria by a halogenated furanone compound.. Microbiology.

[15] Schaber JA, Hammond A, Carty NL, Williams SC, Colmer-Hamood JA (2007). Diversity of biofilms produced by quorum-sensing-deficient clinical isolates of *Pseudomonas aeruginosa*.. J Med Microbiol.

[16] Purevdorj B, Costerton JW, Stoodley P (2002). Influence of hydrodynamics and cell signaling on the structure and behavior of *Pseudomonas aeruginosa* biofilms.. Appl Environ Microbiol.

[17] Heydorn A, Ersboll B, Kato J, Hentzer M, Parsek MR (2002). Statistical analysis of *Pseudomonas aeruginosa* biofilm development: impact of mutations in genes involved in twitching motility, cell-to-cell signaling, and stationary-phase sigma factor expression.. Appl Environ Microbiol.

[18] Brackman G, Cos P, Maes L, Nelis HJ, Coenye T (2011). Quorum sensing inhibitors increase the susceptibility of bacterial biofilms to antibiotics *in vitro* and *in vivo*.. Antimicrob Agents Chemother.

[19] Kolodkin-Gal I, Romero D, Cao S, Clardy J, Kolter R (2010). D-Amino acids trigger biofilm disassembly.. Science.

[20] Davies DG, Marques CN (2009). A fatty acid messenger is responsible for inducing dispersion in microbial biofilms.. J Bacteriol.

[21] Harjai K, Kumar R, Singh S (2010). Garlic blocks quorum sensing and attenuates the virulence of *Pseudomonas aeruginosa*.. FEMS Immunol Med Microbiol.

[22] Smyth AR, Cifelli PM, Ortori CA, Righetti K, Lewis S (2010). Garlic as an inhibitor of *Pseudomonas aeruginosa* quorum sensing in cystic fibrosis–a pilot randomized controlled trial.. Pediatr Pulmonol.

[23] Persson T, Hansen TH, Rasmussen TB, Skinderso ME, Givskov M (2005). Rational design and synthesis of new quorum-sensing inhibitors derived from acylated homoserine lactones and natural products from garlic.. Org Biomol Chem.

[24] Miyamoto CM, Chatterjee J, Swartzman E, Szittner R, Meighen EA (1996). The role of lux autoinducer in regulating luminescence in *Vibrio harveyi*; control of *luxR* expression.. Mol Microbiol.

[25] Bjarnsholt T, Jensen PO, Rasmussen TB, Christophersen L, Calum H (2005). Garlic blocks quorum sensing and promotes rapid clearing of pulmonary *Pseudomonas aeruginosa* infections.. Microbiology.

[26] Christensen LD, van Gennip M, Jakobsen TH, Alhede M, Hougen HP (2012). Synergistic antibacterial efficacy of early combination treatment with tobramycin and quorum-sensing inhibitors against *Pseudomonas aeruginosa* in an intraperitoneal foreign-body infection mouse model.. J Antimicrob Chemother.

[27] Jakobsen TH, van Gennip M, Phipps RK, Shanmugham MS, Christensen LD (2012). Ajoene, a sulfur rich molecule from garlic, inhibits genes controlled by quorum sensing..

[28] Kubec R, Kim S, Musah RA (2002). *S*-Substituted cysteine derivatives and thiosulfinate formation in *Petiveria alliacea*-part II.. Phytochemistry.

[29] Kubec R, Musah RA (2001). Cysteine sulfoxide derivatives in *Petiveria alliacea*.. Phytochemistry.

[30] Musah RA, He Q, Kubec R, Jadhav A (2009). Studies of a novel cysteine sulfoxide lyase from *Petiveria alliacea*: the first heteromeric alliinase.. Plant Physiol.

[31] Block E, Dane AJ, Thomas S, Cody RB (2010). Applications of direct analysis in real time mass spectrometry (DART-MS) in *Allium* chemistry. 2-Propenesulfenic and 2-propenesulfinic acids, diallyl trisulfane *S*-oxide, and other reactive sulfur compounds from crushed garlic and other *Alliums*.. J Agric Food Chem.

[32] Junker LM, Clardy J (2007). High-throughput screens for small-molecule inhibitors of *Pseudomonas aeruginosa* biofilm development.. Antimicrob Agents Chemother.

[33] Kubec R, Kim S, McKeon DM, Musah RA (2002). Isolation of *S*-*n*-butylcysteine sulfoxide and six *n*-butyl-containing thiosulfinates from *Allium siculum*.. J Nat Prod.

[34] Kubec R, Velisek J, Musah RA (2002). The amino acid precursors and odor formation in society garlic (*Tulbaghia violacea* Harv.).. Phytochemistry.

[35] Vanoyan N, Walker SL, Gillor O, Herzberg M (2010). Reduced bacterial deposition and attachment by quorum-sensing inhibitor 4-nitro-pyridine-N-oxide: the role of physicochemical effects.. Langmuir.

[36] Muh U, Schuster M, Heim R, Singh A, Olson ER (2006). Novel *Pseudomonas aeruginosa* quorum-sensing inhibitors identified in an ultra-high-throughput screen.. Antimicrob Agents Chemother.

[37] Parsek MR, Singh PK (2003). Bacterial biofilms: an emerging link to disease pathogenesis.. Annu Rev Microbiol.

[38] Estin ML, Stoltz DA, Zabner J (2010). Paraoxonase 1, quorum sensing, and *P. aeruginosa* infection: a novel model.. Adv Exp Med Biol.

[39] Mulcahy H, Sibley CD, Surette MG, Lewenza S (2011). *Drosophila melanogaster* as an animal model for the study of *Pseudomonas aeruginosa* biofilm infections *in vivo*.. PLoS Pathog.

[40] Nomura YN J, Hayaishi O (1963). *S*-Alkylcysteines: enzymatic cleavage of *S*-methyl-L-cysteine and its sulfoxide.. J Biol Chem.

[41] Altschul SF, Madden TL, Schaffer AA, Zhang J, Zhang Z (1997). Gapped BLAST and PSI-BLAST: a new generation of protein database search programs.. Nucleic Acids Res.

[42] Montazerozohori M, Joohari S, Karami B, Haghighat N (2007). Fast and highly efficient solid state oxidation of thiols.. Molecules.

[43] Krom BP, Cohen JB, McElhaney Feser GE, Cihlar RL (2007). Optimized *candidal* biofilm microtiter assay.. J Microbiol Methods.

[44] Walencka E, Sadowska B, Rozalska S, Hryniewicz W, Rozalska B (2005). Lysostaphin as a potential therapeutic agent for *staphylococcal* biofilm eradication.. Pol J Microbiol.

[45] Kovaleva J, Degener JE, van der Mei HC (2010). Mimicking disinfection and drying of biofilms in contaminated endoscopes.. J Hosp Infect.

[46] Brenner K, Karig DK, Weiss R, Arnold FH (2007). Engineered bidirectional communication mediates a consensus in a microbial biofilm consortium.. Proc Natl Acad Sci U S A.

[47] Apidianakis Y, Rahme LG (2009). *Drosophila melanogaster* as a model host for studying *Pseudomonas aeruginosa* infection.. Nat Protoc.

